# Reduced risks of influenza-associated hospitalization and complications following vaccination among over 2 million older individuals: a nationwide study using target trial emulation framework

**DOI:** 10.1186/s12916-025-03955-w

**Published:** 2025-03-13

**Authors:** Zi-Yang Peng, Yun-Ting Hua, Wan-Ting Huang, Jin-Shang Wu, Huang-Tz Ou

**Affiliations:** 1 Institute of Clinical Pharmacy and Pharmaceutical Sciences, College of Medicine, National Cheng Kung University, Tainan, Taiwan; 2 Global Health Program, College of Public Health, National Taiwan University, Taipei, Taiwan; 3 National Taiwan University Children’s Hospital, Taipei, Taiwan; 4 Department of Family Medicine, National Cheng Kung University Hospital, College of Medicine, National Cheng Kung University Hospital, National Cheng Kung University National Cheng Kung University, Tainan, Taiwan; 5 Department of Family Medicine, National Cheng Kung University Hospital Dou-Liou Branch, College of Medicine, National Cheng Kung University, Yunlin, Taiwan; 6 Department of Family Medicine, College of Medicine, National Cheng Kung University, Tainan, Taiwan; 7 Department of Pharmacy, College of Medicine, National Cheng Kung University, Tainan, Taiwan

**Keywords:** Influenza-associated hospitalizations, Influenza-associated deaths, Infectious/pulmonary diseases, Cardiovascular diseases, Kidney diseases, Influenza vaccination, Target trial emulation

## Abstract

**Background:**

Current evidence on influenza vaccine effectiveness (VE), which is predominately derived from small high-risk older populations and focuses on specific influenza-related complications, might not be generalizable to real-world older populations with diverse characteristics in Taiwan. Therefore, this observational study with a target trial emulation framework aimed to evaluate the clinical effectiveness of an influenza vaccine on influenza infection, complications, and associated healthcare utilization and costs.

**Methods:**

1,214,392 propensity-score-matched pairs of vaccinated and unvaccinated older populations from the influenza season of 2018/2019 were identified from Taiwan’s National Health Insurance Research Database. VE (estimated as 1 minus hazard ratio [HR]*100%) and the HRs were used for influenza events and associated complications, respectively.

**Results:**

Primary analyses show 14% (10–18%) of VE against influenza-associated hospitalization, irrespective of age, frailty status, and underlying influenza risk. Notably, a decline in VE for influenza-associated hospitalization was observed when the observational period following vaccination was extended (25% [19–30%], 23% [18–28%], and 14% [10–18%] for the intervals October 2018–March 2019, October 2018–May 2019, and October 2018–September 2019, respectively). Compared with non-vaccination, having an influenza vaccination significantly reduced risks of influenza-associated death by 30%, various respiratory by 12–26%, cardiovascular complications by 39–47%, and acute kidney injury by 23%. Approximately savings of USD 3,000,000 in total from averting influenza-associated hospitalization following vaccination were found. The non-significant effects of the influenza vaccine on negative control outcomes support the validity of the study procedures.

**Conclusions:**

VE for severe influenza events (i.e., those requiring hospitalization) and related complications among the real-world older population was corroborated. To avoid severe influenza episodes and complications and minimize associated economic consequences, continuous influenza vaccine uptake over different influenza seasons is recommended for this population.

**Supplementary Information:**

The online version contains supplementary material available at 10.1186/s12916-025-03955-w.

## Background

People aged ≥ 65 years are at an increased risk of influenza infection and development of serious influenza-related complications (e.g., cardiovascular [CV], severe pulmonary, and kidney diseases, and death [1]) due to the immunosenescence, multiple chronic health problems, and frailty in this population [1]. In Taiwan, an influenza vaccine is therefore government-funded and strongly recommended to adults aged 65 years or above for reducing influenza transmission as well as averting influenza-associated morbidities and mortalities [2]. Despite these efforts, the uptake rate of the influenza vaccine among the older population in routine clinical practice settings remains suboptimal (e.g., around 50–60% [2, 3]).

Influenza vaccine effectiveness (VE) is commonly reported to inform personal decision-making on vaccination [4,5]. However, there are several caveats regarding current evidence for older populations. First, despite substantial evidence supporting VE in the general older population worldwide from annual epidemiological reports of sentinel surveillance systems [6] and the potential vaccine benefits on avoiding influenza-related complications [7–10], only the subsets (i.e., those with gout [9], disability [11], or breast cancer [12]) or limited numbers (i.e., thousands [7]) of general older populations in Taiwan were analyzed. Therefore, current VE evidence might not be generalizable to general older populations in Taiwan to support decision-making. Second, the concern of residual confounding by indication and healthy vaccinee bias might remain in previous observational studies, with case–control or cohort [5] designs used, leading to imprecise VE and thereby undermining the study validity and confidence to support clinical decision-making [13]. Recently, the target trial emulation framework has been extensively employed in the field of vaccine studies (e.g., COVID-19 vaccine [14]), and this approach comprises an explicit study design framework along with rigorous methodologies which could minimize the possibility of confounding effects and biases commonly seen in the observational studies of real-world data [15]. Lastly, the impact of the uptake of the influenza vaccine on healthcare utilization (e.g., medical costs and length of hospital stay) in Taiwan is also unclear.

Against this background, the present study sought to determine the effectiveness of a standard-dose influenza vaccine on influenza infection, a series of influenza-related complications (i.e., CV, respiratory, and kidney diseases and death), and healthcare utilization and costs in adults aged ≥ 65 years using a target trial emulation approach. Empirical evidence from this study in particular vaccine effectiveness beyond influenza infection (i.e., associated complications and economic consequences) is important for facilitating personal decision-making on vaccination, improving the vaccine uptake rate, and maximizing the value of an influenza vaccine in real-world practice.

## Methods

### Emulation of hypothetical trial using nationwide claims data

This was a retrospective cohort study using a target trial emulation framework. Our study design for the target trial was adapted according to a published randomized, double-blind, clinical trial [16]. The specifications of the target trial are detailed in Additional file 1: Table 1.

Taiwan’s National Health Insurance Research Database (NHIRD) for 2017–2019 was utilized. The National Health Insurance program in Taiwan covers healthcare services (e.g., outpatient, emergency room [ER] visits, hospitalization, and medication prescriptions) for over 99% of Taiwan’s population. Health-related information is therefore longitudinally collected and recorded in the NHIRD. The health records in the NHIRD are individual-level and de-identified. Details of the NHIRD are available elsewhere [17]. This study was approved by the Institutional Review Board of National Cheng Kung University (111–458-2).

### Study population

All individuals in Taiwan aged ≥ 65 years have been eligible for a government-funded influenza vaccine since 2001 [18]. The government-funded vaccine is available each year from October until it runs out or the end of September the following year. Annually, 40% to 60% of older adults received the vaccination; of those vaccinated, > 90% of the vaccinations were administered in October through December. The present study identified all subjects aged ≥ 65 years in the NHIRD for 2018 as the study cohort. Subjects who were first vaccinated in October, November, or December 2018 were placed into the vaccinated group [11, 12]. Those vaccinated in other months (i.e., January to September 2019) or not vaccinated were placed in the unvaccinated group. To emulate the target trial, the eligibility for vaccination was assessed for each older individual (Additional file 1: Table 1). A sequential trial approach [19] (Additional file 1: Fig. S1) with a two-step propensity score (PS) matching was performed to enhance the comparability between the vaccinated and unvaccinated groups regarding baseline characteristics. In the first step, vaccinated subjects were matched with unvaccinated subjects based on age, gender, and city/county in a 1:*n* ratio. A vaccinated subject was matched with multiple unvaccinated subjects (as much as possible) to ensure similar accessibility to healthcare within matched individuals. In the second step, which was performed on the matched pairs in each month as a stratum (i.e., October, November, and December 2018), 1:1 PS-matched pairs of vaccinated and unvaccinated subjects were obtained using 8-to-1-digit greedy matching. Of note, the index date for a vaccinated subject was the date of receiving the influenza vaccine, whereas that for an unvaccinated subject was set to October 15, November 15, or December 15 based on their matched month stratum to minimize the immortal time bias.

Details of the inclusion and exclusion criteria and matching procedure [20] and the operational definitions [21, 22] of the baseline characteristics are given in Additional file 1: Fig. S1 and Table 2. Each study subject was followed from the index date until the occurrence of an influenza event, reception of the influenza vaccine (i.e., the second vaccination for the vaccinated group and the first vaccination for the unvaccinated group), death, or the end of influenza season (i.e., September 30, 2019), whichever came first (i.e., observational analog of the per-protocol scenario).

### Measurements of vaccination status and influenza outcomes

Vaccination status was ascertained from one of the following records in the NHIRD: (1) reception of the influenza vaccine, (2) reimbursement for vaccine administration, or (3) outpatient visit for influenza vaccination, indicating that the influenza vaccination was reimbursed by the Taiwan Center for Disease Control. The main study outcomes were (1) influenza-associated hospitalization, which was defined based on influenza diagnosis codes (the International Classification of Diseases, Tenth Revision, Clinical Modification: J09-J11); (2) influenza-associated outpatient visits, which were defined based on influenza diagnosis codes (J09-J11) with antiviral drug use; and (3) influenza-associated ER visits, which were defined based on influenza diagnosis codes (J09-J11) with antiviral drug use. Because systematic inflammatory responses triggered by influenza events [23] could increase the risks of severe pulmonary [24], CV [25], and kidney diseases [26], these clinical conditions or complications are likely to occur following influenza infection among the older population [27, 28]. The prevention of influenza infection episodes through vaccination is, therefore, crucial to avoid the occurrence of these complications [27, 28]. In this regard, we included influenza-associated complications that occurred during influenza-associated hospitalization as study outcomes of interest. These complications included pneumonia, acute respiratory distress syndrome (ARDS) with ventilator use, sepsis, acute myocardial infarction (AMI), stroke, acute kidney injury, and death, which was ascertained from the Cause of Death files in the NHIRD. Of note, given that the protective effect of a vaccine generally starts 14 days after vaccination [29], study events that occurred ≥ 14 days after the index date were measured. Details of the operational definitions of the study outcomes are available in Additional file 1: Table 3.

### Statistical analysis

The standard mean difference (SMD) was utilized to assess the between-group comparability in patient baseline characteristics. An absolute value of SMD ≥ 0.1 was considered to indicate a statistically significant between-group imbalance. The event rate of a study outcome was estimated as the number of events divided by 1000 person-years. The healthcare costs associated with influenza-associated healthcare utilization, including hospital admissions (and corresponding length of stay, measured in days) and outpatient and ER visits, were estimated from the perspective of the healthcare sector. Costs were standardized into 2022 values using the medical component of the consumer price index in Taiwan and are presented in United States dollars (USD). Student’s *t*-test was used to evaluate the between-group difference in healthcare costs. The Cox proportional hazard model was employed to assess the risk of the study outcome (e.g., influenza infection) with vaccination status. Any unbalanced variables between vaccinated and unvaccinated groups were further treated as covariates and adjusted in the Cox model analysis. The results are presented as hazard ratios (HRs) and associated 95% confidence intervals (CIs). The VE was then calculated as (1 − HR)*100%.

A series of sensitivity analyses were performed to test the robustness of the study findings in the primary analyses. First, to enhance the validity of the measurement of the study outcomes, influenza-associated hospitalization was determined according to the principal diagnosis codes for influenza, and influenza-associated outpatient and ER visits were defined based on the principal diagnosis codes for influenza with the prescription of an antiviral drug. Second, considering influenza occurs throughout the year in Taiwan, the analyses focused on the peak season (month) of influenza, where the end of the follow-up period was restricted to March, April, or May 2019, respectively, to assess whether the VE waned following influenza vaccination [30]. Third, considering the potential of unmeasured confounders (i.e., health awareness), negative control analyses were conducted [31], where influenza events that occurred 7 or 13 days following vaccination, influenza events in the sampled cohort with 50,000 subjects obtained from a vaccine-mismatched season (i.e., influenza season of 2014 or 2015 [32]) using the same patient selection procedures (i.e., the selection of patient cohort followed the target trial emulation framework), and traffic-accident-related hospitalization [30] were treated as study outcomes. No significant risks of these control events with vaccination status were expected (i.e., 95% CI of HR overlapping 1), ensuring the validity of our study materials and procedures. Lastly, given the possibility of the presence of immunosenescence in geriatric populations [33], the modification effect of patients’ baseline characteristics (e.g., age) on vaccination outcomes (i.e., VE) cannot be ruled out. Therefore, a series of interaction tests were carried out for various patient characteristics, including gender (i.e., female or male), age (i.e., ≥ or < 75 years), frailty (i.e., fit or frail), and influenza risk (i.e., presence or absence of high-risk disease conditions such as infectious diseases, blood disorders, and endocrinologic disorders, as specified by Taiwan’s Center for Disease Control), and joint subgroups (i.e., aged ≥ or < 75 years and fit or frail, and aged ≥ or < 75 years and with or without high risk of influenza infection). A two-tailed *p*-value of < 0.05 was considered to indicate a statistically significant difference. All analyses mentioned above were conducted using SAS software version 9.4.

## Results

We identified 3,394,238 subjects aged ≥ 65 years in the NHIRD for 2018 and 2019. After the study eligibility and matching procedures were applied, 1,214,392 pairs of vaccinated and unvaccinated subjects were obtained for the analysis (Additional file 1: Fig. 2). The baseline characteristics of the study cohort before PS matching are provided in Additional file 1: Tables 4–6. Table 1 shows satisfactory between-group comparability in the baseline characteristics (SMD less than 0.1), except for the history of government-funded health examination in the year prior to the index date. In general, the study population had a mean age of 74.3 years and was 46.1% male.

**Table 1:** Baseline characteristics of the overall study population after two-step propensity score matching

Characteristics	After matching		
	Vaccinated subjects	Unvaccinated subjects	SMD^a^
No. of subjects	1,214,392	1,214,392	
**Demographics at index date**			
Age (years, mean ± SD)	74.3 ± 7.3	74.3 ± 7.3	0.00
Male (%)	46.1	46.1	0.00
Influenza infection in prior season (%)	3.7	3.6	0.01
**Multimorbidity frailty category at index date (%)**			
Fit	40.1	43.3	− 0.06
Mild frail	40.5	40.3	0.00
Moderate frail	14.8	12.8	0.06
Severe frail	4.5	3.6	0.05
**Government-funded medical examinations within 1 year before the index date (%)**			
Oral cancer screening	4.1	3.8	0.02
Colorectal cancer screening	16.1	14.5	0.04
Cervical cancer screening	11.1	9.1	0.07
Breast cancer screening	5.5	4.8	0.03
Health examination	36.9	31.0	**0.12**
**Comorbidities within one year before the index date (%)**			
AMI	0.8	0.9	− 0.01
CHF	0.6	0.6	− 0.01
PVD	3.2	3.0	0.01
Cerebrovascular disease	5.4	5.4	0.00
CPD	8.9	7.6	0.05
Rheumatic disease	1.6	1.5	0.01
Peptic ulcer disease	2.1	2.1	0.01
Mild liver disease	10.2	9.3	0.03
Diabetes	31.7	30.9	0.02
Hemiplegia or paraplegia	0.6	0.7	− 0.01
Moderate to severe CKD	11.8	11.1	0.02
Malignancy	8.7	8.9	− 0.01
Moderate or severe liver disease	0.1	0.2	− 0.01
Metastatic solid tumor	0.6	0.9	− 0.03
HIV infection	0.02	0.02	0.00
**Exposure to cardiovascular medications within one year before the index date (%)**			
Antiplatelets	32.4	31.6	0.02
Anticoagulants	5.2	5.7	− 0.02
Cardiac glycosides	1.2	1.3	− 0.01
Antiarrhythmic drugs	5.0	5.0	0.00
Vasodilators	28.8	28.1	0.02
Alpha-blockers	5.7	5.4	0.01
Diuretics	16.3	16.5	− 0.01
Beta-blockers	29.9	30.0	0.00
Calcium channel blockers	37.8	37.4	0.01
RAAS agents	46.0	45.4	0.01
Lipid-lowering agents	40.0	38.2	0.04
**Exposure to pulmonary medications within one year before the index date (%)**			
ICS	0.7	0.6	0.01
SABA	3.9	3.6	0.02
SAMA	1.7	1.7	0.01
LABA	0.6	0.5	0.01
LAMA	1.5	1.2	0.02
SABA and SAMA	0.7	0.7	0.00
LAMA and LABA	1.4	1.2	0.02
LABA and ICS	4.3	3.9	0.02
Theophylline	10.9	9.7	0.04
LTRA	1.4	1.1	0.02
Omalizumab	0.01	0.01	0.00
Systemic corticosteroids	31.3	29.9	0.03
**Monthly premium-based income (per person) at index date (%)**			
≤ 760 USD	31.3	31.0	0.01
760–960 USD	43.0	42.8	0.00
960–1210 USD	6.8	6.9	− 0.01
1210–1527 USD	7.0	7.2	− 0.01
≥ 1,527 USD	12.0	12.1	0.00

*Abbreviations: PSM* propensity score matching, *SMD* standard mean difference, *SD* standard deviation, *AMI* acute myocardial infarction, *CHF* congestive heart failure, *PVD* peripheral vascular disease, *CPD* chronic pulmonary disease, *CKD* chronic kidney disease, *RAAS* renin–angiotensin–aldosterone system, *ICS* inhaled corticosteroid, *SABA* short-acting beta-agonists, *SAMA* short-acting muscarinic antagonist, *LABA* long-acting beta-agonists, *LAMA* long-acting muscarinic antagonist, *LTRA* leukotriene receptor antagonist, *USD* United States dollars

^a^An absolute value of the standard mean difference of ≥ 0.1 indicates a statistically significant difference in baseline characteristics between vaccinated and unvaccinated groups

**Fig. 1:**
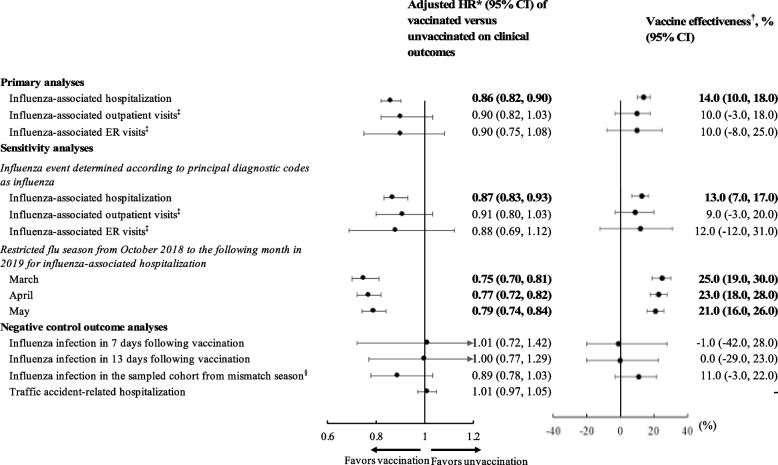
Forest plot of adjusted hazard ratios and associated vaccine effectiveness in primary, sensitivity, and negative control outcome analyses. Abbreviations: HR, hazard ratio; CI, confidence interval; ER, emergency room. *The receipt of government-funded health examinations was unbalanced between groups after a two-step propensity score matching and thus was further adjusted in the Cox model. Bold HRs and 95% CIs indicate statistically significant vaccine effectiveness. ^†^Vaccine effectiveness (VE) = (1-HR)*100%. Bold VE values and 95% CIs indicate a statistically significant vaccine protection effect. ^**‡**^Influenza-associated outpatient or ER visits were defined as having any diagnosis codes of influenza infection and using antiviral drugs. ^§^500,000 subjects were sampled from the entire elderly population in the influenza season of 2014/2015 (which was a vaccine mismatch season), and the same analytic procedures (e.g., the selection of patient cohort based on a target trial emulation framework) were redone in this cohort to estimate the VE

In Fig. 1, the primary analyses show that the event rate of influenza-associated hospitalization was 3.12 and 3.67 per 1000 person-years for vaccinated and unvaccinated subjects, respectively, resulting in a VE of 14% on influenza-associated hospitalization (i.e., adjusted HR [aHR]: 0.86, 95% CI: 0.82–0.90). The event rates of influenza-associated outpatient/ER visits were 0.52/0.20 and 0.56/0.22 for vaccinated and unvaccinated subjects, respectively, but the VE values estimated from these events were insignificant (i.e., VE: 10%/10%, aHRs: 0.90/0.90, 95% CIs: 0.82–1.03/0.75–1.08). The results of sensitivity analyses that assessed influenza events based on principal diagnostic codes and restricted influenza seasons (i.e., October 2018 to the end of March, April, or May 2019) were consistent with the primary analysis findings, showing significant VE for influenza-associated hospitalization (i.e., VE/aHRs [95% CIs]: 13%/0.87 [0.83–0.93], 25%/0.75 [0.70–0.81], 23%/0.77 [0.72–0.82], and 21%/0.79 [0.74–0.84], respectively). No significant effect of influenza vaccination on negative control outcomes was observed (i.e., VE: − 1% [− 42%, 28%] for influenza events that occurred within 7 days following vaccination, 0% [− 29%, 23%] for influenza events within 13 days following vaccination, 11% [− 3%, 22%] for influenza events in the sampled cohort from a mismatched season, and aHR: 1.01 [0.97, 1.05] for traffic accident-related hospitalization). Details of the event rates are given in Additional file 1: Table 7.

**Fig. 2:**
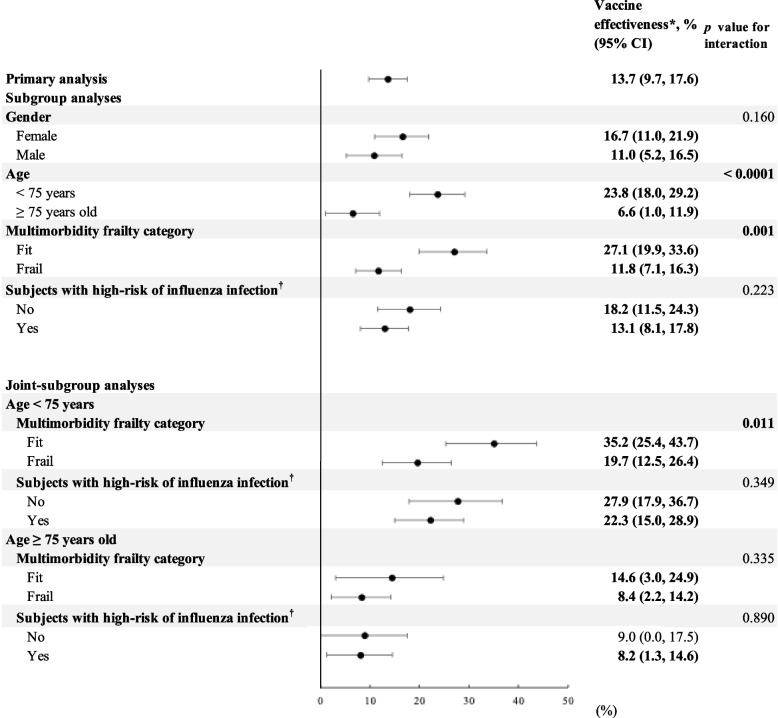
Vaccine effectiveness for influenza hospitalization across subgroup and joint subgroup analyses. Abbreviations: CI, confidence interval. *Vaccine effectiveness (VE) = (1 − HR)*100%. Bold VE values and 95% CIs indicate a statistically significant vaccine protection effect. Details of event rates and hazard ratios of vaccinated versus unvaccinated subjects for influenza hospitalization are available in Additional file 1: Table 7. ^†^According to Taiwan’s Center for Disease Control, individuals with one of the following disease histories determined within one year before the index date were defined as subjects at high risk of influenza infection: infectious diseases, blood disorders, endocrinologic disorders, neurological disorders, cardiovascular diseases, respiratory diseases, digestive system diseases, musculoskeletal system, and connective tissue diseases, urogenital system disease, congenital malformations, and chromosomal abnormalities

Figure 2 shows significant interactions of VE with age and multimorbidity frailty. That is, the VE (95% CIs) for influenza-associated hospitalization was 23% (18–29%) and 11% (5–16%) for subjects aged < and ≥ 75 years, respectively (*p*-value for interaction < 0.0001), and 27% (19–33%) and 11% (7–16%) for fit and frail subjects, respectively (*p*-value for interaction = 0.001). The joint subgroup analyses also indicate significant interaction of vaccine status with multimorbidity frailty in subjects aged < 75 years; i.e., the VE for influenza-associated hospitalization was 35% (25–43%) and 19% (12–26%) for fit and frail subjects, respectively (*p*-value for interaction = 0.011). Details of the event rates and associated aHRs and 95% CIs are given in Additional file 1: Table 8.

Table 2 shows the detailed event rates and associated aHRs (95% CIs) for clinical complications that occurred during influenza hospitalization. Compared with non-vaccination, reception of the influenza vaccine was associated with significantly reduced risks of influenza-associated death (aHR: 0.70 [95% CI: 0.56–0.88]), infectious/pulmonary diseases (i.e., 0.88 [0.81–0.96], 0.85 [0.74–0.98], and 0.34 [0.19–0.61]) for pneumonia, sepsis, and ARDS with ventilator use, respectively), CV diseases (i.e., 0.61 [0.39–0.95] and 0.53 [0.38–0.75] for AMI and stroke, respectively), and kidney diseases (i.e., 0.77 [0.61, 0.96] for acute kidney injury).

**Table 2:** Event rates and associated hazard ratios of vaccination versus non-vaccination for clinical complications that occurred during influenza hospitalization

	No. of events	Event rate (no. of events per 1000 person-years)	No. of events	Event rate (no. of events per 1000 person-years)	Adjusted HR^a^ (95% CI) of vaccinated versus unvaccinated for clinical outcomes	Number needed to be treated
	Vaccinated subjects		Unvaccinated subjects			
Influenza-related deaths	128	0.12	188	0.17	**0.70 (0.56, 0.88)**	26.21
Infectious or pulmonary diseases						
Pneumonia	1052	2.61	1196	3.00	**0.88 (0.81, 0.96)**	39.48
ARDS with ventilator use	15	0.04	44	0.11	**0.34 (0.19, 0.61)**	17.14
Sepsis	383	0.95	454	1.14	**0.85 (0.74, 0.98)**	16.14
Cardiovascular diseases						
Acute myocardial infarction	31	0.08	52	0.13	**0.61 (0.39, 0.95)**	25.00
Stroke	51	0.13	95	0.24	**0.53 (0.38, 0.75)**	12.14
Kidney diseases						
Acute kidney injury	129	0.32	170	0.43	**0.77 (0.61, 0.96)**	14.77

*Abbreviations: HR* hazard ratio, *CI* confidence interval, *ARDS* acute respiratory distress syndrome

^a^The receipt of government-funded health examinations was unbalanced between groups after a two-step propensity score matching and thus was further adjusted in the Cox model

Bold HRs and 95% CIs indicate statistically significant vaccine effectiveness

**Fig. 3:**
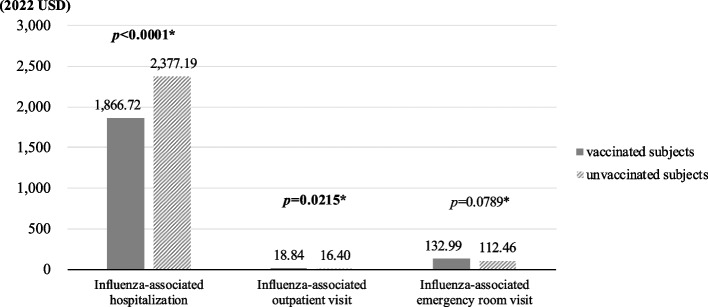
Histogram of influenza-associated healthcare costs per event stratified by vaccine status. *p-values indicate statistical between-group difference in healthcare costs

Figure 3 shows significantly lower influenza-associated hospitalization costs (per admission) (i.e., $1866 versus $2377, *p*-value < 0.0001) and marginally significantly higher influenza-associated outpatient ($18 versus $16, *p*-value = 0.0215) and ER ($132 versus $112, *p*-value = 0.0789) costs (per visit) in vaccinated subjects compared with those for unvaccinated subjects. The results of influenza-associated healthcare costs and length of hospital stay are detailed in Additional file 1: Table 9.

## Discussion

This study of over 2 million older individuals showed the beneficial effect of influenza vaccination on influenza-associated hospitalization and a wide range of related complications, including infectious/pulmonary, CV, and kidney diseases and death. These findings extend the current evidence, which mostly focuses on high-risk older populations, to the general older population in real-world settings. We adopted a target trial emulation framework and a series of sensitivity analyses, which not only enhanced the transparency of study design and procedures but also guaranteed high-quality study results. This strengthens confidence in the reported VE and associated clinical/economic benefits, encouraging individuals to receive an influenza vaccination.

### Comparison of vaccine effectiveness between previous studies and this study

In the present study, for the older population in the influenza season of 2018–2019, the estimated VE values (95% CIs) for influenza-associated hospitalization, outpatient visits, and ER visits were 14% (10%, 18%), 10% (− 3%, 18%), and 10% (− 8%, 25%), respectively, which fall in the range of VE values reported in previous studies (i.e., 12% [− 31%, 40%] [4] to 26% [20%, 31%] [5]). However, caution should be taken when comparing findings across studies due to differences in the influenza viruses circulating worldwide, which are affected by antigenic drift in local or regional geographic areas, operational definitions of influenza events (i.e., laboratory-confirmed influenza [4, 5] versus clinical diagnoses in the present study), and study procedures (i.e., previous cohort [5] or test-negative case–control studies [4] versus the present study using a target trial emulation design) across studies.

In this study, a substantial effort was made (in terms of methodology) to minimize the confounding effects and biases that are commonly seen in studies [4, 5]. First, a series of sensitivity analyses that restricted influenza events to those confirmed by the principal diagnosis were carried out. The results of these analyses were consistent with the primary findings, strengthening confidence in VE in real-world settings and providing clinical insights to facilitate real-world decision-making. Specifically, in these sensitivity analyses, statistically significant protection by influenza vaccination was only shown for influenza-associated hospitalization (i.e., VE [95% CI]: 13% [7%, 17%]), but not for influenza-associated outpatient and ER visits (9% [− 3%, 20%] and 12% [− 12%, 31%], respectively). These findings imply that influenza vaccination may be effective in alleviating the severity of influenza infection (e.g., avert severe cases that require hospitalization), but it does not decrease infection episodes or mild cases (e.g., influenza-associated outpatient and ER visits) [34–36]. Second, we performed several sensitivity analyses using negative control outcomes. The non-significant results supported the success of implementing a target trial emulation design with two-step PS matching in eliminating the concern of unmeasured confounders.

Lastly, considering the possibility of VE attenuation over time following vaccination in older populations [37], sensitivity analyses were conducted to restrict the study follow-up period to different lengths of the peak influenza months in winter (October 2018 to March 2019, October 2018 to April 2019, October 2018 to May 2019, October 2018 to September 2019). It was found that the VE for influenza-associated hospitalization decreased as the time interval increased (i.e., 25% [19%, 30%], 23% [18%, 28%], 21% [16%, 26%], and 14% [10%, 18%] for the considered influenza season lengths, respectively). These results suggest the importance of continuous influenza vaccine uptake over influenza seasons for enhancing immunogenicity against influenza infection in older populations. Also, the provision of high-dose or adjuvant influenza vaccines [38] and the development of a new vaccine platform (e.g., mRNA) [39] are suggested to achieve optimal protection against influenza infection among older individuals.

### Variation of VE for influenza hospitalization by age and multimorbidity frailty in older patients

In this study, aging (which is typically associated with immunosenescence) and frailty were found to be strong effect modifiers. There was a large disparity in the VE values across the subgroups stratified by these two variables. That is, subjects who were younger (i.e., 65 ≤ age < 75 years) or fit in the multimorbidity frailty category had higher VE values compared with those of their counterparties (i.e., aged ≥ 75 years and frail), as supported by significant interaction results (*p* for interaction < 0.05, Fig. 1). These findings were confirmed by interaction tests in the joint subgroup analysis for subjects aged < 75 years and fit or frail. Therefore, these results indicate that the underlying health status (e.g., old age, frailty) may affect VE even in a season with a good match between the vaccine and circulating strains [40, 41]. Young and healthy subjects may have greater VE than those old and frail subjects. Nevertheless, we found that reception of the influenza vaccine was statistically significantly associated with a reduced risk of influenza-associated hospitalization, irrespective of age, frailty status, and with and without high risk of influenza infection; e.g., although low VE values (95% CIs) were obtained for subjects aged 75 years (i.e., 6.6% [1.0%, 11.9%]), frail individuals (11.8% [7.1%, 16.3%]), and high-risk older individuals (13.1% [8.1%, 17.8%]), all estimates were statistically significant. Such specific subgroups in older populations should be prioritized for receiving high-dose, or adjuvanted influenza vaccines whenever those are available in Taiwan. Moreover, the caregivers of these subgroups are recommended to be vaccinated to optimize protection from influenza infection for older individuals through the cocoon strategy [42]. Also, the number needed to treat (NNT) results of influenza vaccination for influenza infection and associated complications can provide explicit insights for clinical decision-making. For example, 47 patients would need to be administered an influenza vaccine relative to non-vaccination for a mean follow-up of 0.8–0.9 years to avert one case having influenza-related hospitalizations (Additional file 1: Table 7). Also, per recommendations from the World Health Organization, a 75% influenza vaccination coverage rate shall be achieved to avoid the annual epidemics, irrespective of age, country, healthcare systems, and race/ethnicity [43]. With consideration of the suboptimal uptake rate of the influenza vaccine in current practice (e.g., 50–60% [2, 3]), promoting the additional benefit of influenza vaccination on the reduction of the CV, pulmonary, and kidney risks to society, adopting high-dose influenza vaccines to the annual vaccination program, and improving vaccine accessibility in rural areas may improve influenza vaccination coverage rates for the general older population, thereby diminishing the disease burden attributable to influenza [40].

### Vaccine effectiveness beyond influenza infection in older patients

In addition to a reduced risk of influenza events, the protective effect of influenza vaccination on a wide spectrum of influenza-related complications (i.e., death and infectious/pulmonary, CV, and kidney diseases) was shown in this study. Previous studies only analyzed the additional benefits of the influenza vaccine among specific subgroups (e.g., patients with gout, CV diseases, or respiratory diseases) of older populations [7–10]. Empirical evidence derived from large-scale general older populations is limited. This study bridges this knowledge gap. It found that influenza vaccination reduces the risks of influenza-related events (i.e., influenza infection, pulmonary disease, and death) by 12–66%, CV diseases by 39–47%, and acute kidney injury by 23%. Mechanisms to support the reduced risks of complications associated with influenza vaccination may be derived from its prevention on influenza infection, which could increase systematic pro-inflammatory cytokines and directly act on vasculature and myocardium, resulting in plaque destabilization and MI or stroke development [44]. Beyond these clinical benefits, the savings from influenza-associated hospitalization following influenza vaccination were also remarkable (i.e., approximately $3,000,000 in total, Additional file 1: Table 9). It is expected that this economic benefit will increase as the uptake rate of the influenza vaccine increases. The savings could be re-allocated to support the universal coverage of the influenza vaccine for the older population and to maintain the national health insurance program.

### Study limitations

First, given the implementation of exclusion criteria according to the study target trial setting, our results may not be generalized to the patients excluded from this study. In particular, a certain number of individuals with dementia (~ 6% of the general older population) were excluded. These individuals are vulnerable to and usually have multiple comorbidities [45], and are thus at high risk for influenza infection and associated complications. Influenza events are likely to be under-recognized due to the decline in cognitive function in these patients, affecting the validity of the VE estimates presented in this study. Hence, to understand the VE in patients with dementia, a prospective pragmatic trial design [46] that relaxes the strict trial patient inclusion criteria to accommodate disadvantaged patients in routine care settings could be adopted in the future. Second, despite a large amount (i.e., over two million) of older subjects included in the current study, a certain proportion of the older population was lost in the process of PS matching. Therefore, in future research, other matching methods (e.g., inverse probability of treatment weighting) can be utilized to strengthen the robustness and generalizability of the current study findings. Third, the present study did not include self-paid influenza vaccination because such data are unavailable. However, given the universal coverage of influenza vaccination for individuals aged ≥ 65 years in Taiwan, this concern might be negligible. Also, potential unmeasured confounders (e.g., health awareness) might exist, which were likely to increase the effect size and thereby lead to underestimated VE. To minimize such a concern, we measured several surrogate indicators (e.g., the receipt of health examination and cancer screenings within 1 year prior to the index date) and adjusted them in analysis (e.g., matching procedures). Fourth, the clinical complications that occurred during influenza hospitalizations (Table 2) were likely affected by the timely receipt of antiviral prescriptions, treatment with antibiotics, and receipt of pneumococcal vaccination, which were not measured and adjusted in our analyses. Fifth, there is a lack of laboratory confirmation (e.g., polymerase chain reaction testing) to identify influenza cases. Lastly, the present study was conducted from the perspective of the healthcare sector and, therefore, did not consider non-medical benefits (e.g., the loss of productivity of family members due to having to care for influenza patients) of influenza vaccination. These additional outcomes following vaccination deserve to be included in a future analysis of the overall benefit of influenza vaccination.

## Conclusions

This empirical study with a large-scale general older population adds supporting evidence regarding the effects of influenza vaccination on severe influenza events (i.e., those requiring hospitalization), influenza-related complications (i.e., infectious/pulmonary, CV, and kidney diseases and death), and potential health care savings. Beneficial effects were found irrespective of individual age, frailty status, and underlying high risk for influenza infection, thereby promoting a wide adoption of the influenza vaccine in this population. To avert severe infection episodes, undesirable complications, and associated economic consequences while maintaining immunogenicity against influenza, the uptake of annual influenza vaccination is recommended for older populations.

## Supplementary Information

Additional file 1: Table 1. Target trial emulation framework. Table 2. Operational definitions of exclusion criteria, baseline characteristics, government-funded medical examinations, and exposure to cardiovascular/pulmonary medications. Table 3. Operational definitions of clinical outcomes. Table 4. Baseline characteristics of the study population identified from October 2018 before the two-step propensity score matching. Table 5. Baseline characteristics of the study population identified from November 2018 before the two-step propensity score matching. Table 6. Baseline characteristics of the study population identified from December 2018 before the two-step propensity score matching. Table 7. Influenza event rates and vaccine effectiveness in primary, sensitivity, and negative control outcome analyses. Table 8. Event rates and hazard ratios of vaccination versus non-vaccination for influenza hospitalization in subgroup and joint subgroup analyses. Table 9. Descriptive results of influenza-associated healthcare resource utilization stratified by status of vaccination. Fig. S1. Study scheme of sequential trial approach. Fig. S2. Flowchart of cohort selection.

## Data Availability

Raw data were generated at Taiwan’s National Health Insurance Research Database. Derived data supporting the findings of this study are available from the corresponding author (Dr. Huang-Tz Ou) on request.

## Abbreviations

ARDS: Acute respiratory distress syndrome
AMI: Acute myocardial infarction
CI: Confidence interval
CV: Cardiovascular
ER: Emergency room
HR: Hazard ratio
NHIRD: National Health Insurance Research Database
NNT: Number needed to treat
PS: Propensity score
SMD: Standard mean difference
USD: United States dollar
VE: Vaccine effectiveness
