# Supplementary material for: Reduced risks of influenza-associated hospitalization and complications following vaccination among over 2 million older individuals: a nationwide study using target trial emulation framework
Source: BMC Med. 2025 Mar 13;23:157. doi: 10.1186/s12916-025-03955-w (PMC11907999; doi:10.1186/s12916-025-03955-w)
Supplement: Supplementary file 1 — Additional file 1: Table 1. Target trial emulation framework. Table 2. Operational definitions of exclusion criteria, baseline characteristics, government-funded medical examinations, and exposure to cardiovascular/pulmonary medications. Table 3. Operational definitions of clinical outcomes. Table 4. Baseline characteristics of the study population identified from October 2018 before the two-step propensity score matching. Table 5. Baseline characteristics of the study population identified from November 2018 before the two-step propensity score matching. Table 6. Baseline characteristics of the study population identified from December 2018 before the two-step propensity score matching. Table 7. Influenza event rates and vaccine effectiveness in primary, sensitivity, and negative control outcome analyses. Table 8. Event rates and hazard ratios of vaccination versus non-vaccination for influenza hospitalization in subgroup and joint subgroup analyses. Table 9. Descriptive results of influenza-associated healthcare resource utilization stratified by status of vaccination. Fig. S1. Study scheme of sequential trial approach. Fig. S2. Flowchart of cohort selection. [file 12916_2025_3955_MOESM1_ESM.docx]

**Additional file 1**

Table 1. Target trial emulation framework

Table 2. Operational definitions of exclusion criteria, baseline characteristics, government-funded medical examinations, and exposure to cardiovascular/pulmonary medications

Table 3. Operational definitions of clinical outcomes

Table 4. Baseline characteristics of the study population identified from October 2018 before the two-step propensity score matching

Table 5. Baseline characteristics of the study population identified from November 2018 before the two-step propensity score matching

Table 6. Baseline characteristics of the study population identified from December 2018 before the two-step propensity score matching

Table 7. Influenza event rates and vaccine effectiveness in primary, sensitivity, and negative control outcome analyses

Table 8. Event rates and hazard ratios of vaccination versus non-vaccination for influenza hospitalization in subgroup and joint subgroup analyses

Table 9. Descriptive results of influenza-associated healthcare resource utilization stratified by status of vaccination

Fig. S1. Study scheme of sequential trial approach

Fig. S2. Flowchart of cohort selection

Table 1. Target trial emulation framework

| **Protocol component** | **Target trial^*^** | **Emulated target trial using real-world data** |
| --- | --- | --- |
| Aim | To assess vaccine effectiveness and influenza-associated healthcare resource utilization among elderly populations with stratification of influenza vaccine status | Same as target trial |
| Eligibility criteria | 1. Subjects aged ≥ 65 years on day of vaccination | Same as target trial |
|  | 1. No influenza vaccination history before beginning of trial | Same as target trial |
|  | 1. No history of Guillain-Barré syndrome, dementia, thrombocytopenia, bleeding disorders, or receiving anticoagulants in the 3 weeks before start of trial | Same as target trial  (We excluded subjects with a history of Guillain-Barré syndrome, dementia, thrombocytopenia, bleeding disorders, alcohol abuse, or drug addiction in the year before October 1, 2018, or exposure to anticoagulants in the 3 weeks before October 1, 2018.) |
|  | 1. No alcohol abuse or drug addiction |  |
|  | 1. No signs and symptoms of acute infectious respiratory illness while receiving vaccine | Similar to target trial  (We assumed that vaccinated subjects had no acute infectious respiratory illnesses or allergic history of influenza vaccine.) |
|  | 1. No systemic hypersensitivity to any vaccine components |  |
| Treatment strategies | Reception of one dose of influenza vaccine or placebo | Same as target trial  (Vaccinated subjects were defined as subjects vaccinated between October and December and unvaccinated subjects were defined as subjects without vaccination or vaccinated in other months [placebo group].) |
| Assignment procedures | Participants randomly assigned to receive influenza vaccine or placebo | Same as target trial  (Two-step propensity score matching was performed to achieve between-group comparability.) |
| Outcomes | Influenza-associated hospitalization, outpatient, emergency room visits, influenza-associated complications, influenza death, and influenza-associated healthcare resource utilization | Same as target trial |
| Follow-up | Participants were followed from randomization until end of flu season. | Same as target trial; the period of study was October 1, 2018, to September 30, 2019. |
| Causal contrasts of interest | Intention-to-treat effect and per-protocol effect | Observational analog of per-protocol effect.  (Subjects who received a second dose of influenza vaccine after follow-up or were vaccinated in other months were censored.) |
| Statistical  analysis plan | Cox model used to obtain hazard ratios with associated 95% confidence intervals for clinical outcomes of interest | Same as target trial |

*Target trial was modified from a published trial (*N Engl J Med.* 2014 Aug 14;371(7):635-45).

Table 2. Operational definitions of exclusion criteria, baseline characteristics, government-funded medical examinations, and exposure to cardiovascular/pulmonary medications

| **Outcomes** | **Operational definitions based on ICD-10-CM diagnosis codes or ATC codes** | **Data files in NHIRD** |
| --- | --- | --- |
| ***Exclusion criteria*** | | |
| Guillain-Barré syndrome | G61.0 | Inpatient, outpatient, and emergency departments |
| Dementia | F01.50, F01.51, F02.81, F03.9, F05, G30.9, G31.01, G31.09, G31.1, G31.83, I69.91 |  |
| Thrombocytopenia | D69 |  |
| Bleeding disorders | D65-D68 |  |
| Alcohol abuse | F10.3-F10.9, F10.0, F10.1, F10.2, G62.1, G31.2, G72.1, I42.6, K29.2, K70.0-K70.4, K70.9, K85.2, K86.0 |  |
| Drug addiction | F11.x–F16.x, F18.x, F19.x, Z71.5, Z72.2 |  |
| Anticoagulants | B01AA03, B01AB01, B01AB04, B01AB05, B01AB06, B01AB10, B01AE07, B01AF01, B01AF02 |  |
| ***Comorbidities in CCI categories*** | | |
| Myocardial infarction | I21.x, I22.x, I25.2 | Inpatient, outpatient, and emergency departments |
| Congestive heart failure | I09.9, I11.0, I13.0, I13.2, I25.5, I42.0, I42.5-I42.9, I43.x, I50.x, P29.0 |  |
| Peripheral vascular disease | I70.x, I71.x, I73.1, I73.8, I73.9, I77.1, I79.0, I79.2, K55.1, K55.8, K55.9, Z95.8, Z95.9 |  |
| Cerebrovascular disease | G45.x, G46.x, H34.0, I60.x-I69.x |  |
| Chronic pulmonary disease | I27.8, I27.9, J40.x-J47.x, J60.x-J67.x, J68.4, J70.1, J70.3 |  |
| Rheumatic disease | M05.x, M06.x, M31.5, M32.x-M34.x, M35.1, M35.3, M36.0 |  |
| Peptic ulcer disease | K25.x-K28.x |  |
| Mild liver disease | B18.x, K70.0-K70.3, K70.9, K71.3-K71.5, K71.7, K73.x, K74.x, K76.0, K76.2-K76.4, K76.8, K76.9, Z94.4 |  |
| Diabetes | E10.x-E14.x |  |
| Hemiplegia or paraplegia | G04.1, G11.4, G80.1, G80.2, G81.x, G82.x, G83.0-G83.4, G83.9 |  |
| Renal disease | I12.0, I13.1, N03.2-N03.7,  N05.2-N05.7, N18.x, N19.x, N25.0, Z49.0-Z49.2, Z94.0, Z99.2 |  |
| Any malignancy, including lymphoma and leukemia, except malignant neoplasm of skin | C00.x-C26.x, C30.x-C34.x,  C37.x-C41.x, C43.x, C45.x-C58.x, C60.x-C76.x, C81.x-C85.x, C88.x, C90.x-C97.x |  |
| Moderate or severe liver disease | I85.0, I85.9, I86.4, I98.2, K70.4, K71.1, K72.1, K72.9, K76.5, K76.6, K76.7 |  |
| Metastatic solid tumor | C77.x-C80.x |  |
| AIDS/HIV | B20.x-B22.x, B24.x |  |
| ***Deficits of mFI to define frailty groups**** | | |
| Disorders of electrolyte and fluid balance | E87 | Inpatient, outpatient, and emergency departments |
| Dementia | F03 |  |
| Sleep disorders and apnea | G47 |  |
| Disorders of eyelids | H02 |  |
| Retinopathy and other eye disorders | H35 |  |
| Glaucoma and ocular hypertension | H40 |  |
| Vertigo or other disorder of vestibular function | H81 |  |
| Hypertension | I10 |  |
| Hypertensive heart disease with/without heart failure | I11 |  |
| Angina | I20 |  |
| Atherosclerotic heart disease and chronic ischemic heart disease | I25 |  |
| Atrial fibrillation and atrial flutter | I48 |  |
| Cardiac arrhythmia | I49 |  |
| Heart Failure | I50 |  |
| Cerebral infarction | I63 |  |
| Cerebral vascular disease | I67 |  |
| Late effect of cerebrovascular diseases | I69 |  |
| Pneumonia | J18 |  |
| Chronic obstructive pulmonary disease | J44 |  |
| Asthma | J45 |  |
| Gastric ulcer | K25 |  |
| Peptic ulcer | K27 |  |
| Functional dyspepsia | K30 |  |
| Constipation | K59 |  |
| Cellulitis | L03 |  |
| Dermatitis | L30 |  |
| Gout | M10 |  |
| Polyosteoarthritis | M15 |  |
| Osteoarthritis | M19 |  |
| Spinal stenosis and spondylopathy | M48 |  |
| Osteoporosis | M81 |  |
| Chronic kidney disease | N18 |  |
| Urinary tract infection | N39 |  |
| Enlarged and nodular prostate | N40 |  |
| Cough | R05 |  |
| Abdominal pain | R10 |  |
| Dizziness and giddiness | R42 |  |
| Presence of functional implant | Z96 |  |
| ***Government-funded medical examination (defined in column “CARD_SEQ_NO”)*** | | |
| Oral cancer screening | IC95 | Inpatient, outpatient, and emergency departments |
| Colorectal cancer screening | IC85 |  |
| Cervical cancer screening | IC31, IC35, IC37 |  |
| Breast cancer screening | IC91 |  |
| Health examination | IC22 |  |
| ***Cardiovascular medication use*** | | |
| Antiplatelets | B01AC | Inpatient, outpatient, and emergency departments |
| Anticoagulants | B01AA, B01AB, B01AE, B01AF |  |
| Cardiac glycosides | C01A |  |
| Antiarrhythmics | C01B |  |
| Vasodilators | C01D, C02D, C02L, C04A |  |
| Alpha blockers | C02CA |  |
| Diuretics | C03 |  |
| Beta blockers | C07 |  |
| Calcium channel blockers | C08 |  |
| RAAS agents | C09 |  |
| Lipid-lowering agents | C10 |  |
| ***Pulmonary medication use*** | | |
| ICS | R03BA | Inpatient, outpatient, and emergency departments |
| SABA | R03AC02, R03AC03, R03AC04, R03AC16 |  |
| SAMA | R03BB01 |  |
| LABA | R03AC12, R03AC13, R03AC18, R03AC19 |  |
| LAMA | R03BB04, R03BB06, R03BB07 |  |
| SABA and SAMA combinations | R03AL01, R03AL02 |  |
| LAMA and LABA combinations | R03AL03, R03AL04, R03AL05, R03AL06, R03AL07, R03AL10 |  |
| LABA and ICS combinations | R03AK04, R03AK05, R03AK07, R03AK08, R03AK09, R03AK10, R03AK11, R03AK12, R03AK13, R03AK14 |  |
| Theophylline | R03DA04 |  |
| LTRA | R03DC |  |
| Omalizumab | R03DX05 |  |

Abbreviations: ICD-10-CM, International Classification of Diseases, Tenth Revision, Clinical Modification; ATC, Anatomical Therapeutic Chemical; NHIRD, National Health Insurance Research Database; CCI, Charlson Comorbidity Index; AIDS, Acquired Immunodeficiency Syndrome; HIV, Human Immunodeficiency Virus; mFI, multimorbidity frailty index; RAAS, renin-angiotensin aldosterone system; ICS, inhaled corticosteroid; SABA, short-acting beta agonists; SAMA, short-acting muscarinic antagonist; LABA, long-acting beta agonists; LAMA, long-acting muscarinic antagonist; LTRA, leukotriene receptor antagonist.

*Frailty categories were determined using the 99th percentile of multimorbidity frailty index (mFI) (ranging between 0 and 1) within the total study population as the upper limit and the associated cut-offs of mFI (i.e., fit: mFI < 0.0658, mild: 0.0658 ≤ mFI < 0.1316, moderate: 0.1316 ≤ mFI < 0.1974, and severe frailty: mFI ≥ 0.1974).

Table 3. Operational definitions of clinical outcomes

| **Outcomes** | **Operational definitions based on ICD-10-CM diagnosis codes, ATC, or order codes** | **Data files in NHIRD** |
| --- | --- | --- |
| Influenza-associated hospitalization | J09, J10, J11 | Inpatient department |
| Influenza-associated outpatient visit | J09, J10, J11 and  J05AH02, J05AH01 | Outpatient department |
| Influenza-associated emergency room visit | J09, J10, J11 and  J05AH02, J05AH01 | Emergency department |
| *Complications during period of influenza-associated hospitalization* | | |
| Influenza-related death | Death dates (ascertained by Cause of Death) were same with discharge date of influenza-associated hospitalization. | Cause of Death and Inpatient department |
| Pneumonia | J12-J18 | Inpatient department |
| ARDS with ventilator use | J80 and  57023B, 57001B, 68036B, 47056B, 47089B |  |
| Sepsis | A40, A41, A207, R6520, R6521, R7881 |  |
| AMI | I21, I22 |  |
| Stroke | I60, I61, I62, I63, I65, I66, I6789 |  |
| AKI | N17 |  |
| *Negative control outcome* | |  |
| Traffic accidence | V00-V99 |  |

Abbreviations: ICD-10-CM, International Classification of Diseases, Tenth Revision, Clinical Modification; ATC, Anatomical Therapeutic Chemical; NHIRD, National Health Insurance Research Database; ARDS, acute respiratory distress syndrome; AMI, acute myocardial infarction; AKI acute kidney injury.

Table 4. Baseline characteristics of the study population identified from October 2018 before the two-step propensity score matching

| Characteristics | Before matching | |  |
| --- | --- | --- | --- |
|  | Vaccinated subjects | Unvaccinated subjects | SMD* |
| No. of subjects | 941,494 | 1,898,669 |  |
| **Demographics at index date** | | | |
| Age (years, mean ± SD) | 74.6 ± 7.3 | 72.9 ± 7.2 | **0.25** |
| Male (%) | 46.5 | 45.8 | **0.25** |
| Influenza infection in prior season (%) | 3.8 | 2.9 | 0.05 |
| **Multimorbidity frailty category at index date (%)** | | | |
| Fit | 38.7 | 57.1 | **-0.37** |
| Mild frailty | 40.9 | 31.4 | **0.20** |
| Moderate frailty | 18.2 | 7.7 | **0.20** |
| Severe frailty | 4.9 | 2.4 | **0.13** |
| **Government-funded medical examinations within one year before index date (%)** | | | |
| Oral cancer screening | 4.5 | 2.9 | 0.08 |
| Colorectal cancer screening | 15.7 | 11.7 | **0.12** |
| Cervical cancer screening | 11.1 | 7.1 | **0.14** |
| Breast cancer screening | 5.2 | 4.1 | 0.05 |
| Health examination | 39.2 | 22.5 | **0.37** |
| **Comorbidities within one year before index date (%)** | | | |
| AMI | 0.8 | 0.8 | 0.00 |
| CHF | 0.6 | 0.5 | 0.01 |
| PVD | 3.4 | 2.3 | 0.06 |
| Cerebrovascular disease | 5.4 | 4.3 | 0.05 |
| Chronic pulmonary disease | 9.2 | 5.6 | **0.14** |
| Rheumatic disease | 1.6 | 1.3 | 0.02 |
| Peptic ulcer disease | 2.2 | 1.6 | 0.05 |
| Mild liver disease | 10.5 | 7.3 | **0.11** |
| Diabetes | 31.9 | 24.9 | **0.16** |
| Hemiplegia or paraplegia | 0.6 | 0.6 | 0.00 |
| Moderate to severe CKD | 12.5 | 8.1 | 0.14 |
| Malignancy | 8.7 | 7.5 | 0.04 |
| Moderate or severe liver disease | 0.1 | 0.2 | 0.00 |
| Metastatic solid tumor | 0.6 | 0.9 | -0.04 |
| HIV infection | 0.02 | 0.01 | 0.01 |
| **Exposure to cardiovascular medications within one year before index date (%)** | | | |
| Antiplatelets | 33.2 | 24.6 | **0.19** |
| Anticoagulants | 5.0 | 4.9 | 0.01 |
| Cardiac glycosides | 1.1 | 1.0 | 0.01 |
| Antiarrhythmic drugs | 5.0 | 4.0 | 0.05 |
| Vasodilators | 29.9 | 21.7 | **0.19** |
| Alpha blockers | 5.9 | 4.0 | 0.09 |
| Diuretics | 16.5 | 13.2 | 0.09 |
| Beta blockers | 30.2 | 24.4 | **0.13** |
| Calcium channel blockers | 38.4 | 30.3 | **0.17** |
| RAAS agents | 46.6 | 36.8 | **0.20** |
| Lipid-lowering agents | 40.2 | 30.6 | **0.20** |
| **Exposure to pulmonary medications within one year before index date (%)** | | | |
| ICS | 0.6 | 0.4 | 0.03 |
| SABA | 3.9 | 2.7 | 0.07 |
| SAMA | 1.6 | 1.4 | 0.02 |
| LABA | 0.6 | 0.4 | 0.04 |
| LAMA | 1.5 | 0.9 | 0.06 |
| SABA and SAMA | 0.7 | 0.5 | 0.02 |
| LAMA and LABA | 1.4 | 0.8 | 0.06 |
| LABA and ICS | 4.3 | 2.9 | 0.08 |
| Theophylline | 11.2 | 7.4 | **0.13** |
| LTRA | 1.4 | 0.8 | 0.05 |
| Omalizumab | 0.01 | 0.01 | 0.01 |
| Systemic corticosteroids | 31.9 | 24.4 | **0.17** |
| **Monthly premium-based income (per person) at index date (%)** | | | |
| ≤ 760 USD | 30.2 | 35.4 | **-0.11** |
| 760-960 USD | 45.4 | 37.3 | **0.17** |
| 960-1,210 USD | 6.4 | 7.2 | -0.03 |
| 1,210-1,527 USD | 6.5 | 7.8 | -0.05 |
| ≥ 1,527 USD | 11.4 | 12.2 | -0.03 |

Abbreviations: PSM, propensity score matching; SMD, standard mean difference; SD, standard deviation; AMI, acute myocardial infarction; CHF, congestive heart failure; PVD, peripheral vascular disease; CPD, chronic pulmonary disease; CKD, chronic kidney disease; RAAS, renin-angiotensin aldosterone system; ICS, inhaled corticosteroid; SABA, short-acting beta agonists; SAMA, short-acting muscarinic antagonist; LABA, long-acting beta agonists; LAMA, long-acting muscarinic antagonist; LTRA, leukotriene receptor antagonist; USD, United States dollars.

*An absolute value of the standard mean difference of ≥ 0.1 indicates a statistically significant difference in baseline characteristics between vaccinated and unvaccinated groups.

Table 5. Baseline characteristics of the study population identified from November 2018 before the two-step propensity score matching

| Characteristics | Before matching | |  |
| --- | --- | --- | --- |
|  | Vaccinated subjects | Unvaccinated subjects | SMD* |
| No. of subjects | 250,323 | 1,898,669 |  |
| **Demographics at index date** | | | |
| Age (years, mean ± SD) | 73.8 ± 7.3 | 72.9 ± 7.2 | **0.12** |
| Male (%) | 46.4 | 45.8 | **0.12** |
| Influenza infection in prior season (%) | 3.6 | 2.9 | 0.04 |
| **Multimorbidity frailty category at index date (%)** | | | |
| Fit | 41.7 | 56.9 | **-0.31** |
| Mild frailty | 39.9 | 31.5 | **0.18** |
| Moderate frailty | 14.1 | 9.1 | **0.16** |
| Severe frailty | 4.3 | 2.5 | **0.10** |
| **Government-funded medical examinations within one year before index date (%)** | | | |
| Oral cancer screening | 3.6 | 2.8 | 0.04 |
| Colorectal cancer screening | 16.2 | 11.5 | **0.14** |
| Cervical cancer screening | 11.0 | 7.1 | **0.14** |
| Breast cancer screening | 5.9 | 4.1 | 0.09 |
| Health examination | 33.8 | 22.6 | **0.25** |
| **Comorbidities within one year before index date (%)** | | | |
| AMI | 0.9 | 0.8 | 0.00 |
| CHF | 0.6 | 0.5 | 0.00 |
| PVD | 2.9 | 2.3 | 0.04 |
| Cerebrovascular disease | 5.6 | 4.4 | 0.06 |
| Chronic pulmonary disease | 9.1 | 5.6 | **0.13** |
| Rheumatic disease | 1.7 | 1.3 | 0.03 |
| Peptic ulcer disease | 2.1 | 1.6 | 0.04 |
| Mild liver disease | 9.8 | 7.3 | 0.09 |
| Diabetes | 31.5 | 25.0 | **0.15** |
| Hemiplegia or paraplegia | 0.7 | 0.6 | 0.02 |
| Moderate to severe CKD | 10.8 | 8.2 | 0.09 |
| Malignancy | 9.0 | 7.7 | 0.05 |
| Moderate or severe liver disease | 0.1 | 0.2 | 0.00 |
| Metastatic solid tumor | 0.7 | 1.0 | -0.03 |
| HIV infection | 0.03 | 0.01 | 0.01 |
| **Exposure to cardiovascular medications within one year before index date (%)** | | | |
| Antiplatelets | 31.7 | 24.7 | **0.16** |
| Anticoagulants | 5.6 | 5.0 | 0.03 |
| Cardiac glycosides | 1.2 | 1.1 | 0.01 |
| Antiarrhythmic drugs | 5.1 | 4.1 | 0.05 |
| Vasodilators | 27.3 | 21.8 | **0.13** |
| Alpha blockers | 5.7 | 4.0 | 0.08 |
| Diuretics | 16.3 | 13.4 | 0.08 |
| Beta blockers | 29.6 | 24.5 | **0.11** |
| Calcium channel blockers | 37.2 | 30.4 | **0.14** |
| RAAS agents | 45.7 | 37.0 | **0.18** |
| Lipid-lowering agents | 40.1 | 30.7 | **0.20** |
| **Exposure to pulmonary medications within one year before index date (%)** | | | |
| ICS | 0.7 | 0.4 | 0.04 |
| SABA | 4.2 | 2.8 | 0.08 |
| SAMA | 1.9 | 1.4 | 0.04 |
| LABA | 0.6 | 0.4 | 0.04 |
| LAMA | 1.6 | 0.9 | 0.06 |
| SABA and SAMA | 0.8 | 0.5 | 0.03 |
| LAMA and LABA | 1.4 | 0.9 | 0.05 |
| LABA and ICS | 4.7 | 3.0 | 0.09 |
| Theophylline | 11.0 | 7.4 | **0.12** |
| LTRA | 1.5 | 0.9 | 0.06 |
| Omalizumab | 0.02 | 0.01 | 0.01 |
| Systemic corticosteroids | 30.6 | 24.6 | **0.13** |
| **Monthly premium-based income (per person) at index date (%)** | | | |
| ≤ 760 USD | 33.7 | 35.4 | -0.04 |
| 760-960 USD | 37.6 | 37.3 | 0.01 |
| 960-1,210 USD | 7.4 | 7.2 | 0.01 |
| 1,210-1,527 USD | 7.9 | 7.8 | 0.00 |
| ≥ 1,527 USD | 13.4 | 12.2 | 0.03 |

Abbreviations: PSM, propensity score matching; SMD, standard mean difference; SD, standard deviation; AMI, acute myocardial infarction; CHF, congestive heart failure; PVD, peripheral vascular disease; CPD, chronic pulmonary disease; CKD, chronic kidney disease; RAAS, renin-angiotensin aldosterone system; ICS, inhaled corticosteroid; SABA, short-acting beta agonists; SAMA, short-acting muscarinic antagonist; LABA, long-acting beta agonists; LAMA, long-acting muscarinic antagonist; LTRA, leukotriene receptor antagonist; USD, United States dollars.

*An absolute value of the standard mean difference of ≥ 0.1 indicates a statistically significant difference in baseline characteristics between vaccinated and unvaccinated groups.

Table 6. Baseline characteristics of the study population identified from December 2018 before the two-step propensity score matching

| Characteristics | Before matching | |  |
| --- | --- | --- | --- |
|  | Vaccinated subjects | Unvaccinated subjects | SMD* |
| No. of subjects | 49,934 | 1,898,669 |  |
| **Demographics at index date** | | | |
| Age (years, mean ± SD) | 73.2 ± 7.1 | 72.9 ± 7.2 | 0.04 |
| Male (%) | 47.4 | 45.8 | 0.04 |
| Influenza infection in prior season (%) | 3.7 | 2.9 | 0.04 |
| **Multimorbidity frailty category at index date (%)** | | | |
| Fit | 41.9 | 56.8 | **-0.30** |
| Mild frailty | 39.6 | 31.5 | **0.17** |
| Moderate frailty | 14.2 | 9.2 | **0.16** |
| Severe frailty | 4.3 | 2.5 | **0.10** |
| **Government-funded medical examinations within one year before index date (%)** | | | |
| Oral cancer screening | 4.1 | 2.8 | 0.07 |
| Colorectal cancer screening | 17.2 | 11.4 | **0.16** |
| Cervical cancer screening | 10.9 | 7.1 | **0.13** |
| Breast cancer screening | 6.1 | 4.0 | 0.09 |
| Health examination | 34.3 | 22.9 | **0.26** |
| **Comorbidities in one year before index date (%)** | | | |
| AMI | 0.9 | 0.9 | 0.00 |
| CHF | 0.6 | 0.6 | 0.00 |
| PVD | 2.7 | 2.3 | 0.03 |
| Cerebrovascular disease | 5.2 | 4.4 | 0.04 |
| Chronic pulmonary disease | 8.8 | 5.6 | **0.12** |
| Rheumatic disease | 1.6 | 1.3 | 0.03 |
| Peptic ulcer disease | 2.0 | 1.6 | 0.03 |
| Mild liver disease | 10.1 | 7.3 | **0.10** |
| Diabetes | 31.6 | 25.1 | **0.14** |
| Hemiplegia or paraplegia | 0.8 | 0.6 | 0.02 |
| Moderate to severe CKD | 10.9 | 8.3 | 0.09 |
| Malignancy | 8.6 | 7.7 | 0.03 |
| Moderate or severe liver disease | 0.2 | 0.2 | 0.00 |
| Metastatic solid tumor | 0.6 | 1.0 | -0.04 |
| HIV infection | 0.03 | 0.01 | 0.01 |
| **Exposure to cardiovascular medications within one year before index date (%)** | | | |
| Antiplatelets | 31.4 | 24.7 | **0.15** |
| Anticoagulants | 5.7 | 5.1 | 0.03 |
| Cardiac glycosides | 1.2 | 1.1 | 0.01 |
| Antiarrhythmic drugs | 5.4 | 4.2 | 0.06 |
| Vasodilators | 27.2 | 21.9 | **0.12** |
| Alpha blockers | 5.5 | 4.1 | 0.07 |
| Diuretics | 15.6 | 13.5 | 0.06 |
| Beta blockers | 30.0 | 24.6 | **0.12** |
| Calcium channel blockers | 36.6 | 30.5 | **0.13** |
| RAAS agents | 45.6 | 37.2 | **0.17** |
| Lipid-lowering agents | 40.6 | 30.9 | **0.20** |
| **Exposure to pulmonary medications within one year before index date (%)** | | | |
| ICS | 0.8 | 0.4 | 0.04 |
| SABA | 4.2 | 2.9 | 0.07 |
| SAMA | 1.9 | 1.5 | 0.03 |
| LABA | 0.6 | 0.4 | 0.03 |
| LAMA | 1.5 | 0.9 | 0.06 |
| SABA and SAMA | 0.8 | 0.6 | 0.03 |
| LAMA and LABA | 1.5 | 0.9 | 0.06 |
| LABA and ICS | 4.5 | 3.1 | 0.07 |
| Theophylline | 11.0 | 7.4 | **0.12** |
| LTRA | 1.5 | 0.9 | 0.06 |
| Omalizumab | 0.0 | 0.01 | 0.01 |
| Systemic corticosteroids | 31.6 | 24.8 | **0.15** |
| **Monthly premium-based income (per person) at index date (%)** | | | |
| ≤ 760 USD | 31.5 | 35.4 | -0.08 |
| 760-960 USD | 41.2 | 37.3 | 0.08 |
| 960-1,210 USD | 7.2 | 7.2 | 0.00 |
| 1,210-1,527 USD | 7.7 | 7.8 | 0.00 |
| ≥ 1,527 USD | 12.4 | 12.2 | 0.00 |

Abbreviations: PSM, propensity score matching; SMD, standard mean difference; SD, standard deviation; AMI, acute myocardial infarction; CHF, congestive heart failure; PVD, peripheral vascular disease; CPD, chronic pulmonary disease; CKD, chronic kidney disease; RAAS, renin-angiotensin aldosterone system; ICS, inhaled corticosteroid; SABA, short-acting beta agonists; SAMA, short-acting muscarinic antagonist; LABA, long-acting beta agonists; LAMA, long-acting muscarinic antagonist; LTRA, leukotriene receptor antagonist; USD, United States dollars.

*An absolute value of the standard mean difference of ≥ 0.1 indicates a statistically significant difference in baseline characteristics between vaccinated and unvaccinated groups.

Table 7. Influenza event rates and vaccine effectiveness in primary, sensitivity, and negative control outcome analyses

|  | No. of events | Event rate (no. of events per 1,000 person-years) | No. of events | Event rate (no. of events per 1,000 person-years) | Adjusted HR* (95% CI) of vaccinated versus unvaccinated on clinical outcomes | Vaccine effectiveness^†^ (95% CI) | Numbers needed to treated |
| --- | --- | --- | --- | --- | --- | --- | --- |
|  | Vaccinated subjects | | Unvaccinated subjects | |  |  |  |
| **Primary analyses** | | | | | | |  |
| Influenza-associated hospitalization | 3,445 | 3.12 | 4,001 | 3.67 | **0.86 (0.82, 0.90)** | **14 (10, 18)** | 46.79 |
| Influenza-associated outpatient visits**^‡^** | 571 | 0.52 | 609 | 0.56 | 0.90 (0.82, 1.03) | 10 (-3, 18) | 54.96 |
| Influenza-associated ER visits**^‡^** | 223 | 0.20 | 245 | 0.22 | 0.90 (0.75, 1.08) | 10 (-8, 25) | 77.82 |
| **Sensitivity analyses** | | | | | | |  |
| *Influenza event determined according to principal diagnostic codes as influenza* | | | | | | |  |
| Influenza-associated hospitalization | 2,231 | 2.02 | 2,556 | 2.34 | **0.87 (0.83, 0.93)** | **13 (7, 17)** | 27.23 |
| Influenza-associated outpatient visits**^‡^** | 477 | 0.43 | 515 | 0.47 | 0.91 (0.80, 1.03) | 9 (-3, 20) | 48.92 |
| Influenza-associated ER visits**^‡^** | 124 | 0.11 | 138 | 0.13 | 0.88 (0.69, 1.12) | 12 (-12, 31) | 67.01 |
| *Restricted flu season from October 2018 to the following month in 2019 for influenza-associated hospitalization* | | | | | | |  |
| March | 1,220 | 2.39 | 1,645 | 3.24 | **0.75 (0.70, 0.81)** | **25 (19, 30)** | 9.26 |
| April | 1,444 | 2.37 | 1,904 | 3.15 | **0.77 (0.72, 0.82)** | **23 (18, 28)** | 10.41 |
| May | 1,747 | 2.46 | 2,238 | 3.18 | **0.79 (0.74, 0.84)** | **21 (16, 26)** | 12.69 |
| **Negative control outcome analyses** | | | | | | |  |
| Influenza infection in 7 days following vaccination | 67 | 0.06 | 66 | 0.06 | 1.01 (0.72, 1.42) | -1 (-42, 28) | -1,449.81 |
| Influenza infection in 13 days following vaccination | 113 | 0.10 | 115 | 0.11 | 1.00 (0.77, 1.29) | 0 (-29, 23) | 141.48 |
| Influenza infection in the sampled cohort from mismatch season^§^ | 381 | 2.58 | 424 | 2.91 | 0.89 (0.78, 1.03) | 11 (-3, 22) | 47.69 |
| Traffic accident-related hospitalization | 4,860 | 4.40 | 4,673 | 4.29 | 1.01 (0.97, 1.05) | - | -332.52 |

Abbreviations: HR, hazard ratio; CI, confidence interval; ER, emergency room.

*Reception of government-funded health examination was unbalanced between groups after two-step propensity score matching and thus further adjusted in Cox model. Bold HRs and 95% CIs indicate statistically significant vaccine effectiveness.

^†^Vaccine effectiveness (VE) = (1-HR)*100%. Bold VE values and 95% CIs indicate a statistically significant vaccine protection effect.

**^‡^**Influenza-associated outpatient or ER visits were defined as having any diagnosis codes of influenza and using antiviral drugs.

^§^500,000 subjects were sampled from the entire older population in the influenza season of 2014/2015 (which was a vaccine mismatch season), and the same analytic procedures (e.g., the selection of patient cohort followed the target trial emulation framework) were redone in this cohort to estimate VE.

Table 8. Event rates and hazard ratios of vaccination versus non-vaccination for influenza hospitalization in subgroup and joint subgroup analyses

|  | No. of subjects | | Estimated event rate (events/1,000 person-years) | | Estimated HR associated with vaccinated versus unvaccinated subjects* (95% CI) |
| --- | --- | --- | --- | --- | --- |
|  | Vaccinated | Unvaccinated | Vaccinated | Unvaccinated |  |
| **Primary analysis** | 1,214,392 | 1,214,392 | 3.12 | 3.67 | 0.86 (0.82, 0.90) |
| ***Subgroup analyses*** | | | | | |
| **Gender** | | | | | |
| Female | 654,221 | 654,221 | 2.75 | 3.34 | 0.83 (0.78, 0.89) |
| Male | 560,171 | 560,171 | 3.55 | 4.06 | 0.89 (0.84, 0.95) |
| **Age** | | | | | |
| < 75 years | 684,323 | 684,323 | 2.01 | 2.65 | 0.76 (0.71, 0.82) |
| ≥ 75 years | 530,069 | 530,069 | 4.58 | 5.01 | 0.93 (0.88, 0.99) |
| **Multimorbidity frailty category** | | | | | |
| Fit | 487,146 | 525,800 | 1.62 | 2.24 | 0.73 (0.66, 0.80) |
| Frail | 727,246 | 688,592 | 4.13 | 4.78 | 0.88 (0.84, 0.93) |
| **Subjects with high risk of influenza infection**^†^ | | | | | |
| No | 569,124 | 594,643 | 2.13 | 2.65 | 0.82 (0.76, 0.89) |
| Yes | 645,268 | 619,749 | 3.99 | 4.67 | 0.87 (0.82, 0.92) |
| ***Joint subgroup analyses*** | | | | | |
| **Age < 75 years** | | | | | |
| **Multimorbidity frailty category** | | | | | |
| Fit | 321,151 | 328,647 | 1.08 | 1.67 | 0.65 (0.56, 0.75) |
| Frailty | 363,172 | 355,676 | 2.83 | 3.57 | 0.80 (0.74, 0.88) |
| **Subjects with high risk of influenza infection**^†^ | | | | | |
| No | 332,236 | 335,324 | 1.27 | 1.77 | 0.72 (0.63, 0.82) |
| Yes | 352,087 | 348,999 | 2.70 | 3.50 | 0.78 (0.71, 0.85) |
| **Age ≥ 75 years** | | | | | |
| **Multimorbidity frailty category** | | | | | |
| Fit | 165,995 | 197,153 | 2.67 | 3.21 | 0.85 (0.75, 0.97) |
| Frail | 364,074 | 332,916 | 5.47 | 6.11 | 0.92 (0.86, 0.98) |
| **Subjects with high risk of influenza infection**^†^ | | | | | |
| No | 236,888 | 259,319 | 3.36 | 3.80 | 0.91 (0.83, 1.00) |
| Yes | 293,181 | 270,750 | 5.58 | 6.20 | 0.92 (0.85, 0.99) |

Abbreviations: HR, hazard ratio; CI, confidence interval.

*Reception of government-funded health examination was unbalanced between groups after two-step propensity score matching and thus further adjusted in Cox model. Bold HRs and 95% CIs indicate statistically significant vaccine effectiveness.

^†^According to Taiwan’s Center for Disease Control, individuals with one of the following disease histories determined within one year before the index date were defined as subjects at a high risk of influenza: infectious diseases, blood disorders, endocrinologic disorders, neurological disorders, cardiovascular diseases, respiratory diseases, digestive system diseases, musculoskeletal system and connective tissue diseases, urogenital system disease, congenital malformations, and chromosomal abnormalities.

Table 9. Descriptive results of influenza-associated healthcare resource utilization stratified by status of vaccination

|  | Vaccination status | Number of events | Q1 | Q2 | Q3 | Mean | Minimum | Maximum | Standard deviation | Total |
| --- | --- | --- | --- | --- | --- | --- | --- | --- | --- | --- |
| Length of hospital stay per admission (days) | Vaccinated | 3,445 | 4 | 6 | 10 | 9.64 | 1 | 338 | 12.95 |  |
|  | Unvaccinated | 4,001 | 4 | 6 | 12 | 10.21 | 1 | 257 | 12.63 |  |
| *Influenza-associated healthcare costs (per admission or visit), presented in 2022 USD* | | | | | | | | | | |
| Hospitalization | Vaccinated | 3,445 | 468.70 | 820.30 | 1,718.61 | 1,866.72 | 52.62 | 196,723.30 | 4,673.40 | 6,430,841.00 |
|  | Unvaccinated | 4,001 | 480.62 | 866.22 | 1,985.97 | 2,377.19 | 49.04 | 112,617.88 | 5,058.84 | 9,511,155.24 |
| Outpatient visit | Vaccinated | 571 | 11.04 | 15.17 | 21.69 | 18.84 | 1.75 | 188.84 | 18.94 | 10,757.01 |
|  | Unvaccinated | 609 | 9.79 | 14.87 | 20.16 | 16.40 | 1.65 | 208.33 | 13.97 | 9,990.46 |
| Emergency room visit | Vaccinated | 223 | 67.13 | 107.24 | 155.83 | 132.99 | 20.10 | 693.01 | 105.56 | 29,656.61 |
|  | Unvaccinated | 245 | 56.42 | 96.33 | 132.57 | 112.46 | 22.77 | 427.53 | 76.67 | 27,551.75 |

Fig. S1. Study scheme of sequential trial approach

^*^The location (i.e., city/county) of a study subject was determined as follows: for vaccinated subjects, the location was the place where they received the influenza vaccine; for unvaccinated subjects, the location was the place of their residence as ascertained from the Registry for Beneficiary file in the National Health Insurance Research Database.

^†^The propensity score (PS) of receiving the influenza vaccine was conditioned on the baseline characteristics of each subject and calculated using a logistic regression analysis. The patient baseline characteristics included demographics (i.e., age, gender), history of influenza infection, a series of comorbidities (e.g., cardiovascular [CV] and kidney diseases), exposure to CV and pulmonary medications, and a multimorbidity frailty category, all measured from the year before the index date. In addition, as suggested by clinical experts, monthly premium-based income at the index date and the history of receipt of government-funded medical examinations (i.e., the screening of oral, colorectal, cervical, and breast cancers and health examinations) within one year before the index date were measured as surrogate indicators for social-economic status and health-seeking behaviors, respectively, and adjusted in the logistic regression analysis for PS estimation.

Fig. S2. Flowchart of cohort selection

Individuals aged ≥ 65 years in October 2018/September 2019 influenza season identified from NHIRD (N=3,394,238)

Exclusion^*^ of subjects having any one of the following conditions in year before October 1, 2018 (N=253,818):

1. Guillain- Barré syndrome (n=359)
2. Dementia (n=211,374)
3. Thrombocytopenia (n=18,602)
4. Bleeding disorder (n=3,354)
5. Drug addiction (n=1,266)
6. Alcohol abuse (n=11,400)
7. Recent use of anticoagulants (within 21 days) before October 31, 2018 (n=13,646)

Vaccinated subjects (N=1,241,751)

Unvaccinated subjects (N=1,898,669)

Two-step propensity-score-matched^†^ pairs of vaccinated and unvaccinated subjects (N=1,214,392)

Abbreviations: NHIRD, National Health Insurance Research Database.

^*^To mitigate the potential confounding effect from the co-existence of infection risks, Subjects with one of the following prior conditions were excluded: 1) Guillain-Barré syndrome, 2) dementia, 3) thrombocytopenia, 4) bleeding disorder, 5) alcohol abuse, or 6) drug addiction within one year before October 1, 2018, or 7) exposure to anticoagulants within 21 days before October 1, 2018.

^†^Details of matching procedure were as follows. To enhance the comparability between the vaccinated and unvaccinated groups in terms of baseline characteristics, two-step propensity score (PS) matching was performed. In the first step, vaccinated subjects were matched with unvaccinated subjects based on age, gender, and city/county in a 1:n ratio. That is, a vaccinated subject was matched with multiple unvaccinated subjects (as much as possible) to ensure similar accessibility to healthcare within matched individuals. Specifically, the location (i.e., city/county) of a study subject was determined as follows: for vaccinated subjects, the location was the place where they had received the influenza vaccine; for unvaccinated subjects, the location was their place of residence ascertained from the Registry for Beneficiary file in the NHIRD. In the second step, which was performed on the matched pairs in each month as a stratum (i.e., October, November, and December 2018), 1:1 PS-matched pairs of vaccinated and unvaccinated subjects were obtained using 8-to-1-digit greedy matching. Of note, the index date for a vaccinated subject was the date of receiving the influenza vaccine, whereas that for an unvaccinated subject was set to October 15, November 15, or December 15 based on their matched month stratum. The PS (probability) of receiving the influenza vaccine was conditioned on the baseline characteristics of each subject and calculated using a logistic regression analysis. The patient baseline characteristics included demographics (i.e., age, gender, influenza history), a series of comorbidities (e.g., CV and kidney diseases), exposure to CV and pulmonary medications, and a multimorbidity frailty category, all measured from the year before the index date. In addition, as suggested by clinical experts, monthly premium-based income at the index date and the history of government-funded medical examinations (i.e., the screening of oral, colorectal, cervical, and breast cancers and health examinations) within one year before the index date were measured as surrogate indicators for social-economic status and health-seeking behaviors, respectively, and taken into account in the logistic regression analysis for PS estimation.
